# Pharmacological effects of triamcinolone associated with surgical glue on cutaneous wound healing in rats

**DOI:** 10.1590/acb399624

**Published:** 2024-12-06

**Authors:** Rosana Soares Araújo Doci, Filipe Feitosa de Carvalho, Rodrigo César Gomes, Reinaldo José Gianini, Camilla Fanelli, Irene de Lourdes Noronha, Nelson Brancaccio dos Santos, Moema de Alencar Hausen, Daniel Komatsu, Priscila Randazzo-Moura

**Affiliations:** 1 Pontifícia Universidade Católica de São Paulo – Faculty of Medical and Health Sciences – Program of Postgraduate in Biomaterials and Regenerative Medicine – São Paulo (SP) – Brazil.; 2 Pontifícia Universidade Católica de São Paulo – Faculty of Medical and Health Sciences – Biomaterials Laboratory – São Paulo (SP) – Brazil.; 3 Universidade de São Paulo – Medical School – Laboratory of Cellular, Genetic, and Molecular Nephrology – São Paulo (SP) – Brazil.; 4 Pontifícia Universidade Católica de São Paulo – Faculty of Medical and Health Sciences – Pathology Laboratory – São Paulo (SP) – Brazil.

**Keywords:** Adhesives, Adrenal Cortex Hormones, Cyanoacrylates, Biocompatible Materials

## Abstract

**Purpose:**

The surgical glue is widely used in closing cutaneous surgical wounds. Corticosteroids are indicated for their anti-inflammatory and immunomodulatory properties. The present work evaluated the pharmacological effects of triamcinolone (AT) incorporated into surgical glue (C) on the initial phase of the wound healing process in Wistar rats.

**Methods:**

Through *in-vivo* studies, the effects of the healing process, C or C+AT in the same rat were evaluated for seven and 14 days post-surgery.

**Results:**

The C+AT association did not change the physicochemical properties of the polymer. This association in wound healing confirmed the anti-inflammatory and immunomodulatory effects of the corticosteroid, with less neovascularization and fibrosis, in addition to the remodeling of the extracellular matrix carried out by the balance of myofibroblasts and less dense collagen fibers, culminating in tissue regeneration and possible reduction of side effects.

**Conclusion:**

This association is a powerful and innovative pharmacological tool, promising in translational medicine.

## Introduction

Surgery is essential for the management of several conditions that affect health, and its importance in the context of global public health is undeniable, since evidence suggests that 11% of years of life lost, adjusted for disability, are correctable with surgery^1^. In this context, the skin is the first and last organ to be addressed in a surgical procedure, requiring post-operative care in order to avoid complications such as dehiscence and local infection.

The cyanoacrylate adhesive glue used as an alternative for closing surgical incisions is a chemical substance with bactericidal activity, synthesized in 1949, but it was only in the 1960s that it began to be used in surgical procedures^2^. Surgical glue (2-octyl-cyanoacrylate) quickly polymerizes in an exothermic reaction and bonds to the most superficial epithelium of the skin, forming a water barrier over the approximate edges of the wound to allow uninterrupted healing, producing a crust over the surface in which it was applied, keeping the tissues interconnected. Among its advantages, the practicality of its execution and the need to return to remove the suture thread stand out. The aesthetic results, however, are controversial, and local complications have been also described, including dehiscence and contact dermatitis when compared to traditional sutures^3^^-^ 6.

A pharmacological alternative to prevent unsightly scars are corticosteroids, in particular triamcinolone, which can be administered locally to the surgical incision to prevent the accumulation of seroma in the serous fluid^7^. Experiments conducted by Burusapat et al.^8^ concluded that immediate injection of triamcinolone acetonide is also an option for treating keloids, associated with a lower recurrence rate and no complications.

The search for biomaterials associated with drugs has grown exponentially, and clinical evidence is often lacking, which justifies the originality and importance of this study in analyzing the association of a corticosteroid (triamcinolone) incorporated with 2-octyl-cyanoacrylate surgical glue (*in-vitro* and *in-vivo* studies) in a pre-clinical trial, evaluating the existence of changes in the initial phase of healing of intradermal surgical wounds, reducing the inflammatory process, but without interfering in the final healing process, in addition to promoting fewer post-surgical complications, such as dehiscence, contact dermatitis and inadequate increase in collagen formation causing unsightly scars.

## Methods

The commercial surgical glue, 2-octyl-cyanoacrylate (C), was acquired from the Company Johnson & Johnson, brand Dermabond Ethicon. The triamcinolone acetonide (AT) in powder form was donated by the Phito Formulas Company, Sorocaba, São Paulo, Brazil.

### Release test

Controlled release of triamcinolone was realized. Samples were individually immersed in 6 mL of phosphate-buffered saline (PBS) solution (0,01 M) and kept in thermostatic bath at 37°C. Aliquots (3 mL) were collected and replaced with the same volume (3 mL) in a time interval from 0 to 120 h. The release of arnica was performed in a ultraviolet (UV) spectrophotometer (Femto Cirrus 80, São Paulo, Brazil) at 274 nm. The quantification of triamcinolone released was calculated using the mean obtained via a duplicate, as compared to a standard curve containing known concentrations.

### Fourier transform infrared spectroscopy

Fourier transform infrared spectroscopy (FTIR) spectra were recorded by the Perkin Elmer Spectrum 65 Fourier transform infrared spectrometer with an ATR cell. Spectra over 4,000–500-cm^-1^ range were obtained with 64 scans and a resolution of 4 cm-1.

### Thermogravimetric analysis

The thermal properties of membranes were studied using the thermogravimetric analysis (TA Instruments, New Castle, DE, United States of America) (model TGA55). Samples with a mass of approximately 10 mg were analyzed. The thermogravimetric analysis was carried out at the temperature from 25 to 700°C with the heating rate equal to 10°C•min-1 under the nitrogen atmosphere (10 mL·min^-1^).

### Differential scanning calorimetry

The differential scanning calorimetry (DSC) analysis was performed using a DSC 250 instrument (TA Instruments). The DSC parameters used in this analysis were: heating rate of 10°C·min^-1^, nitrogen atmosphere and temperature range of 25 to 250°C. The samples had approximately 7 mg.

### Scanning electron microscopy

The samples were fixed in Karnovsky solution (2.5% glutaraldehyde, 2% paraformaldehyde in 0.2 M sodium cacodylate buffer, 18 uM calcium chloride and pH 7.4) at 4°C for at least 2 hours, subjected to treatment to assume electro density with 1% osmium tetroxide for 1 h. Then, dehydration was carried out in increasing series of ethanol until the concentration of 100% and drying equivalent to critical point using increasing series of ethanol and hexamethyldisilazane (HDMS) until final drying at room temperature containing only HDMS. Finally, gold plasma coverage was carried out at the thickness of 40 nm, followed by mounting in a sample holder for scanning electron microscopy (SEM) and analyzed using a JEOL JSM-6490LV analytical scanning electron microscope under a voltage of 5 kV using secondary electrons.

### In-vivo assays

The animals used were male Wistar rats, aged approximately 3 months, weighing between 200 and 250 g, provided from Bioterium of the Medical and Health Sciences Faculty at Pontifícia Universidade Católica de São Paulo, under ethical committee approval number 2022/132. They were divided into two groups:

- Contralateral treatment (seven days): on the left side of the back only surgical glue (C) and on the right side of the back surgical glue + 20 mg/mL of acetonide of triamcinolone (C+AT);
- Contralateral treatment (14 days): on the left side of the back only surgical glue (C) and on the right side of the back surgical glue + 20 mg/mL of acetonide of triamcinolone (C+AT);

Intact skin samples from the back of some animals were collected and considered the *naïve* group, for contralateral comparison of possible histological changes in the experimental groups. For each study group, eight to ten animals were assigned, and the wounds were analyzed seven and 14 days after the surgical intervention. For surgery, the animals were anesthetized with ketamine and xylasin intraperitoneally. After trichotomy and antisepsis of the animal’s back, two surgical incisions (3 cm each) were made. These wounds were parallel and separated from each other by 4 cm and depth of 2 mm. The biomaterial (C or C+AT) was applied directly to the incision line, with the edges held bidigitally in approximation for 60 seconds. In seven or 14 days, all animals were euthanized with a lethal dose of inhaled isoflurane and tissues from both incisions were biopsied for histological and immunohistochemical analyses.

### Histology analyses: hematoxylin and eosin and Masson’s trichrome staining

Samples of scar tissue from the surgical incisions were collected on the seventh and 14th day. Skin biopsies were embedded in paraffin blocks after fixation in buffered formalin solution. The embedded skin tissues were cut into 5-μm thick sections using a microtome (Leica, RM 2245, United States of America) and collected on glass slides. Subsequently, the sections were deparaffinized and stained with hematoxylin and eosin (HE) or Masson’s trichrome (MT). The stained histological sections were observed under a light microscope (Eclipse E800, Nikon) and evaluated in random order, a double-blind study. In HE staining, the following parameters were evaluated: fibrosis, angiogenesis, presence of lymphocytes and plasma cells, presence of macrophages, and neutrophils; with MT, collagen fibers were identified. For both stainings, the score (0–3) was used: 0 = absent; 1 = light; 2 = moderate; and 3 = intense.

### Immunohistochemical analysis

Immunohistochemical analysis was performed through the indirect immunoperoxidase technique. The CD68 surface marker was employed to detect macrophages, while the positivity for alpha-Smooth Muscle Actin (a-SMA) was used to identify myofibroblasts. We further evaluated the positivity of fibronectin in the studied samples. Briefly, after deparaffinization, antigen retrieval was performed with 10mM Citric Acid, pH 6.0, at 95ºC, in a microwave oven for 15 minutes. Slides were washed in Tris buffer saline (TBS) solution (pH 7.6), underwent endogenous peroxidase blocking (Peroxidase Block #ACA015, ScyTek Laboratories, United States of America) and nonspecific staining blocking (Super Block #AAA125, ScyTek Laboratories, United States of America). For the detection of macrophages, the samples were incubated with 50 L of monoclonal mouse anti-CD68 clone ED-1 primary antibody solution (Serotec, #MCA341R), diluted [1:200]. For the identification of myofibroblasts, sections were incubated with 50 mL of monoclonal mouse anti-a-SMA primary antibody solution (Sigma, #A5228), at [1:1,000]. Finally, to identify fibronectin, we used the primary antibody polyclonal rabbit anti-fibronectin (Sigma, #F3648), at [1:200]. The antibodies were diluted in 1% bovine serum albumin (BSA) solution, and incubations were carried out in humid chambers, at 4°C, overnight.

After this period, slides were washed in TBS again and incubated with ENVISION Flex Mouse & Rabbit HRP peroxidase-labeled secondary antibody solution (Dako #K4061) for 45 minutes. Sequentially, sections were once again washed in TBS and subjected to a Dab-based solution (diaminobenzidine, dab chromogen + substrate kit #ACB030, ScyTek Laboratories, United States of America), followed by counterstaining with Harrys’ hematoxylin. All parameters (macrophages, myofibroblasts, fibronectin) were observed under a light microscope (Eclipse E800, Nikon), evaluated in random order, a double-blind study and stratified (score 0–3): 0 = absent; 1 = light; 2 = moderate; and 3 = intense, according to the changes found.

### Statistical analysis

Statistical analysis was performed using the WSAnova command of the STATA program, which performed analysis of variance (ANOVA) for repeated measures between dependent samples. The mean, standard deviation, Snedecor F- and p-value were described, as well as the number of counts and rats in each statistical test. Differences were considered statistically significant when *p* < 0.05.

## Results and Discussion

### Release test

**Figure 1:**
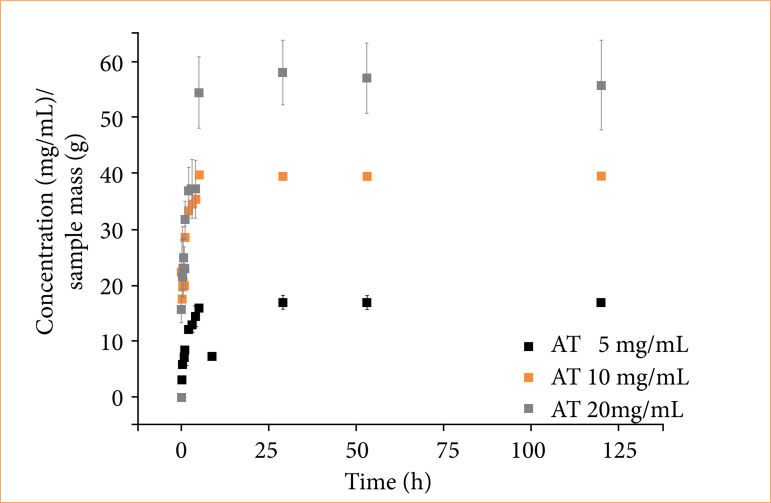
Data of controlled release assays with surgical glue loaded to triamcinolone acetonide (AT).

Source: Elaborated by the authors.

This is an *in-vitro* analysis used to evaluate the release profile of the sample of interest and choose the concentration of corticosteroid to be incorporated into the glue, so that the release of the drug occurs in approximately five days, sufficient time for the corticosteroid acts on the inflammation process, without interfering with final healing^9^. It can be observed in Fig. 1 that the sample containing 5 mg/mL of AT exhibited the lowest amount of released AT. This result is consistent, given that it had the lowest AT concentration. Conversely, the sample with 20 mg/mL of AT demonstrated the highest amount of released AT, due to its higher AT concentration. Hence, the result depicted in Fig. 1 indicates that, the higher the amount of AT incorporated into the glue (C), the greater the amount of AT released into the medium.

The tested samples showed biphasic release patterns with an initial burst of AT release followed by sustained release after 30 hours. The initial burst release is influenced by the molecules available on the surface of the sample, whereas sustained release is affected by both the molecules in the sample and those bound to it. Thus, obtained data pointed out a fast initial release rate of AT with values of 4.12, 7.76, and 8.91 mg·h^-1^ for samples with 5, 10 and 20 mg/mL of AT, respectively (Fig. 1). As anticipated, the sample with the highest concentration of AT demonstrated the fastest release, consistent with its greater availability of AT molecules on the surface. So, when applied to treat lesions, it is crucial that the molecules are rapidly released into the surrounding environment.

### Fourier transform infrared spectroscopy

**Figure 2:**
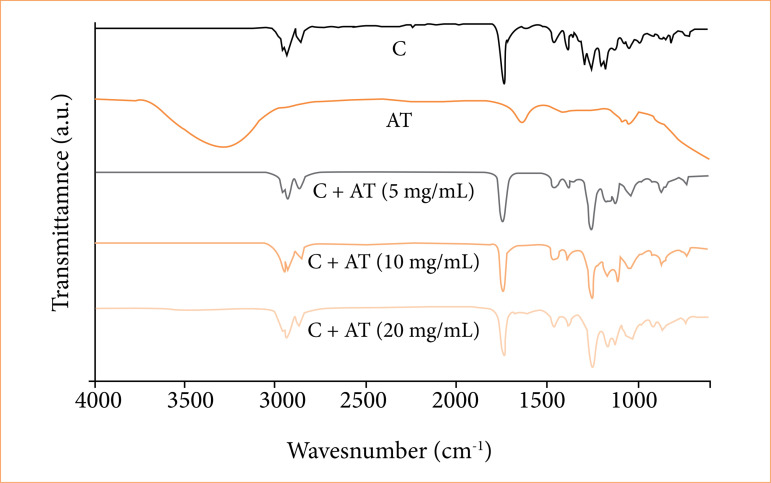
Fourier transform infrared spectroscopy spectra of only glue **(C)**, only triamcinolone acetonide (AT) and C+AT with different amounts of AT.

Source: Elaborated by the authors.

The pure glue film presented a peak at 3,120 cm^-1^ related to the stretching of the =CH2 bond; 2,930 cm^-1^ referring to the stretching of the -CH_3_ bond; 1,732 cm^-1^ referring to the stretching of the C=O bond, and 1,250 cm^-1^ referring to the stretching of the C-O-C bond. The pure AT presents a peak between 3,500 and 3,000 cm^-1^ attributed to the stretching of the O-H bond; a peak at 1,704 cm^-1^ referring to carbonyl stretching (C=O); a peak at 1,662 cm^-1^ attributed to the C=C bond; and a peak at 1,050 cm^-1^ referring to the vibrational stretching of the C-F bond present10 (Fig. 2).

In the glue films with varying amounts of AT, the appearance of new peaks was not observed following the addition of AT to the glue. This suggests the absence of a chemical bond between the glue and AT molecules, which is a desirable characteristic for facilitating the release of AT molecules.

Additionally, following the addition of AT to pure glue at various concentrations, the spectra of the formulations remained nearly identical to those of the pure glue film, showing no evidence of the presence of the AT of any concentration. A possible explanation for this phenomenon is associated with the low quantity of AT present in the formulation, which may be situated in the dry glue film rather than on its surface, thereby complicating its identification. However, in the glue film with 20 mg/mL of AT, peaks at 1,704 and 1,662 cm^-1^ were observed, indicating the presence of AT molecules on the surface of the dry glue film. This observation supports the explanation that at contents below 10 mg of AT these molecules are located inside the dry glue film due to their low concentration.

Thermogravimetric analysis

**Figure 3:**
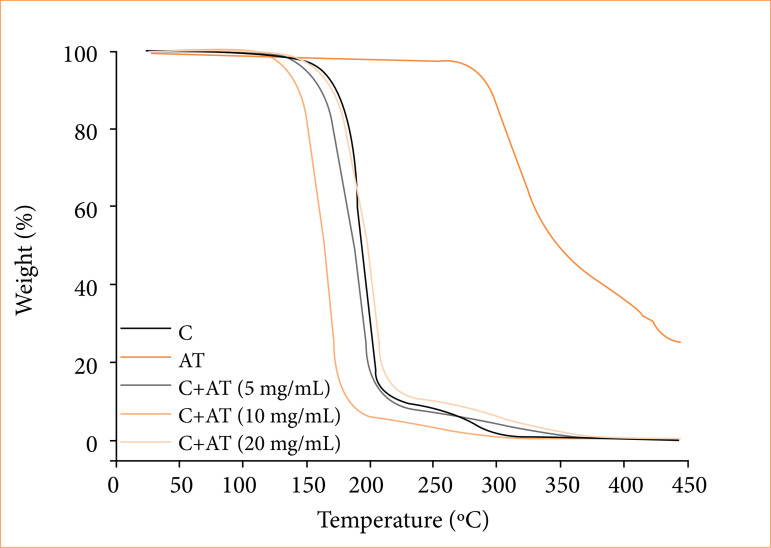
Thermogravimetric curves of only glue **(C)**, only triamcinolone acetonide (AT) and C+AT with different amounts of AT.

Source: Elaborated by the authors.

In this experiment, it was possible to observe that the pure glue film showed a mass loss close to 200°C (Fig. 3). The octyl-2-cyanoacrylate begins its thermal degradation between 150 and 200°C, with total degradation occurring before 250°C. In turn, the pure AT powder showed a mass loss close to 300°C, which is related to its degradation (Fig. 3). According to Dandamudi et al.^10^, AT showed mass loss close to 290°C. Glue films with 5 and 10 mg/mL showed lower thermal stability than pure glue film. In this case, glue film with 10 mg/mL showed the lowest thermal stability among all the samples analyzed. However, glue film with 20 mg/mL showed practically the same thermal stability than pure glue film (Fig. 3).

**Figure 4:**
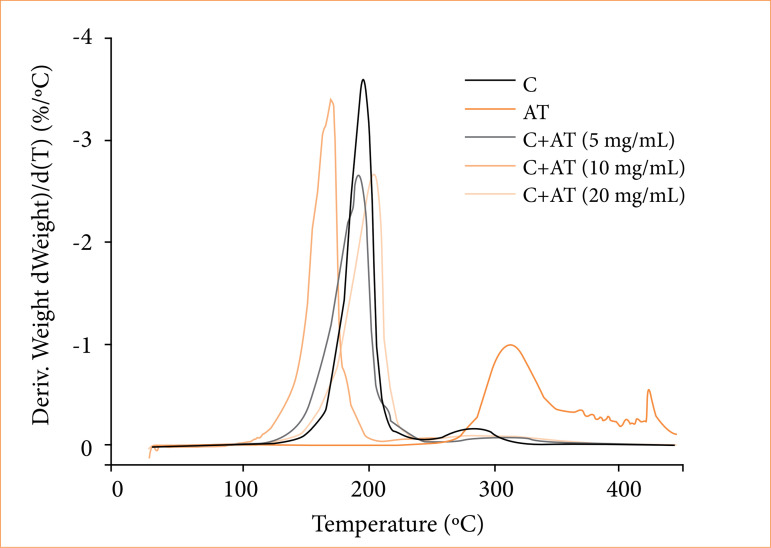
Differential thermogravimetry curves of only glue **(C)**, only triamcinolone acetonide (AT) and C+AT with different amounts of AT.

Source: Elaborated by the authors.

The differential thermogravimetry (DTG) analysis revealed a decrease in the peak height of the glue films with different amounts of AT compared to the pure glue film. Although glue films with different amounts of AT exhibited higher mass loss than pure glue film, the DTG analysis (Fig. 4) revealed a decrease in the peak height of the glue films with different amounts of AT compared to the pure glue film. This decreasing is more pronounced to glue films with 5 and 20 mg/mL of AT. This indicates a lower thermal degradation rate of glue films with 5 and 20 mg/mL of AT than pure glue film. The observed behavior can be attributed to an increase in the interaction between compounds of films, AT, and glue molecules^11^.

### Differential scanning calorimetry

**Figure 5:**
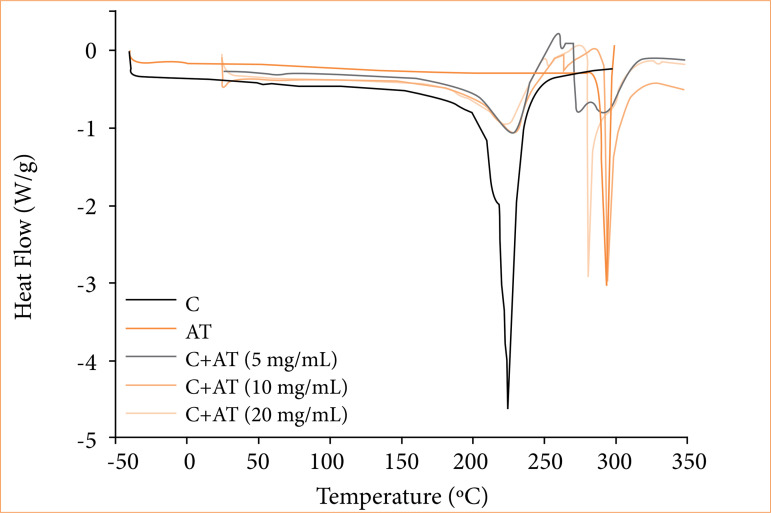
Differential scanning calorimetry curves of only glue (C), only triamcinolone acetonide (AT) and C+AT with different amounts of AT (Exo up).

Source: Elaborated by the authors.

Figure 5 illustrates the differential scanning calorimetry (DSC) curves of the pure glue film, that showed a peak at 225°C. This temperature is close to what was found on the thermogravimetric analysis, in the region of great mass loss. Therefore, this endothermic peak can be attributed to the degradation of the pure glue film. In turn, the pure AT powder presented a peak at 290°C. It is related to the degradation of AT sample, since this temperature is in the region of great mass loss on the thermogravimetric analysis curve (Fig. 3).

In the DSC curve of the glue film with AT, two endothermic peaks were observed. The first peak occured at 225°C, and the second peak at 280°C (Fig. 5). In this case, the first peak is related to the degradation of the pure glue film, and the second peak to the degradation of the AT^12^.

### Scanning electron microscopy

**Figure 6:**
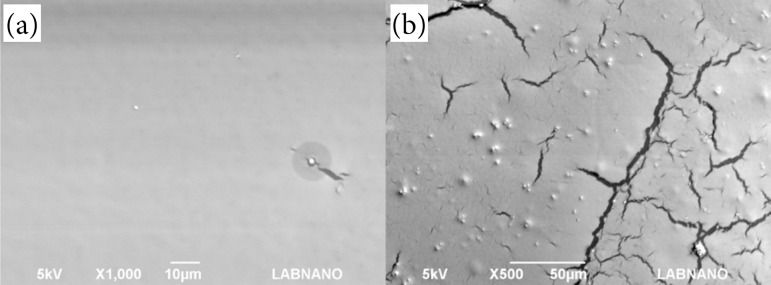
Scanning electron microscopy images of **(a)** glue film and **(b)** glue film with triamcinolone acetonide (20 mg/mL).

Source: Elaborated by the authors.

The pure glue film showed the homogeneous, smooth, and uniform surface (Fig. 6). Hu et al.^13^ also demonstrated that docetaxel-loaded cholesterol-PEG-co-modified poly (n-Butyl) cyanoacrylate nanoparticles presented a surface close to the one seen in this work.

The glue film with 20 mg/mL of AT showed a heterogeneous surface, that is, this sample presented a cracked surface (Fig. 6). It happened because there was an interruption of glue molecular cohesion due to the presence of AT. The AT molecule appears in the form of aggregates and has low solubility^14^. However, when associated with glue, there is the formation of agglomerates, which are dispersed on the glue film. Along the same lines, Yao et al.^15^ studied biodegradable nanoparticles of poly(butyl-2-cyanoacrylate), a polymeric surgical glue associated with sodium dodecyl sulfate, evaluated the morphological transitions, and showed that the addition of new active ingredients in tissue adhesive polymers can interfere with the characteristics of the area of tridimensional surface identified by SEM.

### Histology analyses: hematoxylin and eosin and Masson’s trichrome staining

The presence of fibrosis was significantly lower in C+AT (14 days, Table 1). This lower amount of fibrosis suggests better healing, especially in the remodeling phase, with consequent closure of the wound closer to the complete skin. Corticosteroids decrease the synthesis of collagen and glycosaminoglycans, reducing the inflammatory process in the wound, promoting control of fibroblasts and increasing hypoxia^16^.

**Table 1:** Histological analysis of slides stained in HE, in the different experimental groups of scar tissue.

Parameters/Time		Experimental Groups					
		C			C+AT		*p*-value
		Mean	SD		Mean	SD	
**Fibrosis**							
	7 days	1.88	0.73		1.80	0.63	0.87
	14 days	2.00	0.88		1.61	0.94	0.01*
**Neovascularization**							
	7 days	2.17	0.91		1.86	0.65	0.01*
	14 days	1.31	1.08		1.17	1.03	0.35
**Lymphocytes/Plasmocytes**							
	7 days	2.00	0.90		1.95	0.73	0.63
	14 days	1.05	0.91		1.27	1.06	0.11
**Macrophages**							
	7 days	1.85	1.03		1.76	0.98	0.76
	14 days	0.92	0.91		1.18	0.97	0.04*
**Neutrophils**							
	7 days	1.79	0.97		2.26	0.70	0.01*
	14 days	1.05	0.98		1.17	1.06	0.40

C: surgical glue; AT: surgical glue + triamcinolone acetonide; 0: absent; 1: light; 2: moderate; 3: intense;

* *p* < 0.05 when compared to the glue group (contralateral);

SD: standard deviation.

Source: Elaborated by the authors.

Neovascularization was significantly lower in C+AT (seven days, Table 1), since corticosteroids act by causing the production of inflammatory cytokines, such as vascular endothelial growth factor, transforming growth factor beta and interleukin-1^17^. The intensity and final angiogenic response appear to be linked to wound healing (Table 1). Therefore, both decreased inflammation and decreased neovascularization are characteristics of optimal healing and reduced scar formation^18^.

The presence of neutrophils was significantly higher in C+AT (seven days), which is in line with the fact that these cells are the first line in the healing process, with a higher concentration 24 hours after the injury, and are attracted by chemotactic substances released by platelets. Neutrophils adhere to the epithelial wall by binding to selectins (endothelial membrane receptors) and cytokine lesions that aid in bacterial destruction, in the initial healing process, and are gradually replaced by macrophages^19^^,^ 20, also corroborating the increased presence of macrophages in the C+AT treatment (14 days). The increased expressions of neutrophils at seven days and macrophages at 14 days suggested a positive outcome of the healing process, as both release cytokines and anti-inflammatory mediators that allow the healing process to be regulated (Table 1).

**Figure 7:**
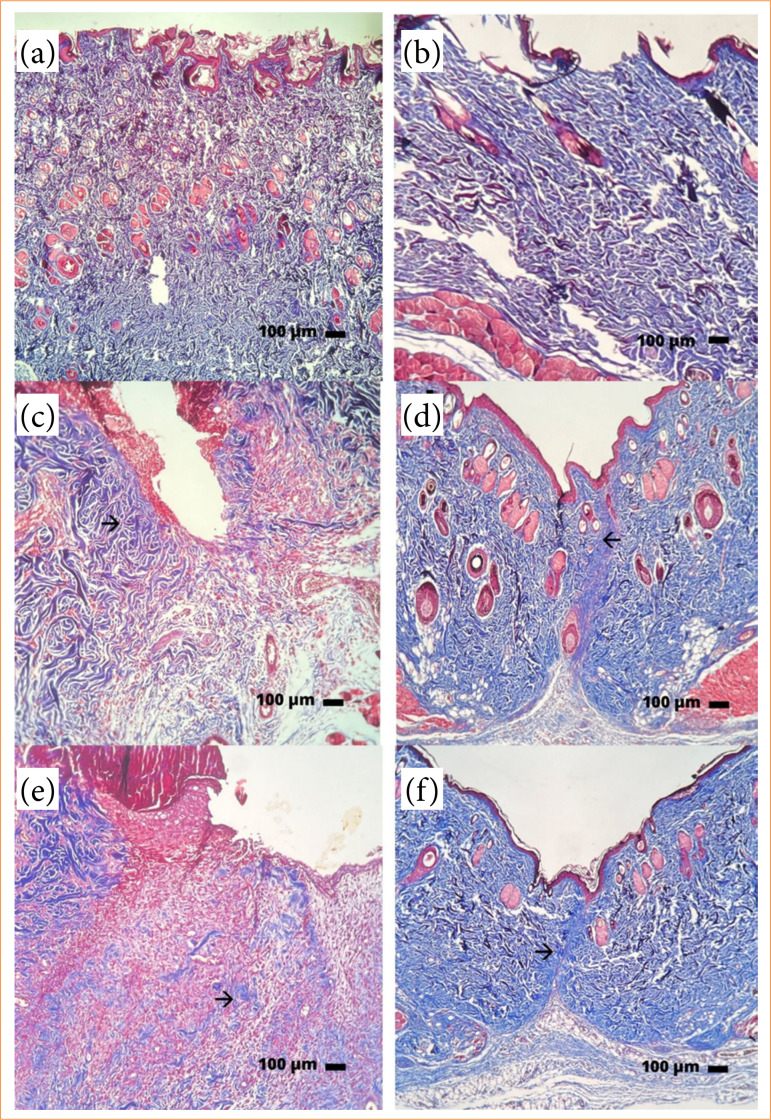
Masson’s trichrome histological panel of scar tissue from Wistar rats from different experimental groups for seven or 14 days. **(a)** Näive seven days; **(b)** Näive 14 days; **(c)** C seven days; **(d)** C 14 days; **(e)** C+AT seven days; **(f)** C+AT 14 days.

When evaluating collagen fibers, there was no significant difference between treatments at seven and 14 days, however, it was possible to observe an increase in the quantity of collagen fibers (Table 2). The fibers appeared organized and without increasing thickness, indicating a regulatory action of the released corticosteroid, controlling the inflammatory process, and, consequently, preventing the formation of disorganized collagen fibers (Fig. 7). According to Kauh et al.^21^, post-surgical responses that received triamcinolone injection after removal of the keloid expressed reduction in type-I collagen, compared to untreated skin, indicating a negative regulation of the expression of this collagen gene.

**Table 2:** Histological analysis of slides stained in Masson’s trichrome, in the different experimental groups of scar tissue.

Parameters/Time		Experimental Groups					
		C			C+AT		*p*-value
		**Mean**	**SD**		**Mean**	**SD**	
**Collagen fibers**							
	7 days	1.71	0.50		1.86	0.52	0.14
	14 days	1.78	0.96		1.85	0.78	0.64

C: surgical glue; AT: surgical glue + triamcinolone acetonide; 0: absent; 1: light; 2: moderate; 3: intense;

* *p* < 0.05 when compared to the glue group (contralateral);

SD: standard deviation.

Source: Elaborated by the authors.

C: glue; AT: triamcinolone acetonide; →collagen fibers; 200X magnification; Bar: 100 μm. Source: Elaborated by the authors.

### Immunohistochemical analysis

Traditionally, CD68 is explored as an important cytochemical marker to immunostain monocytes/macrophages in histochemical analysis of inflamed tissues, tumor tissues, and other immunohistopathological applications^22^. In this analysis, no statistically significant result was evidenced, but an increase in macrophages was shown in the C+AT treatment (seven and 14 days, Table 3). Data in the literature show that a deficient response of macrophages causes damage to the healing and closure of wounds, as the expressive participation of macrophages lead to fibrotic tissue and efficient tissue repair, in which resident macrophages can replicate and increase in number and orchestrate wound healing treatments^23^^,^ 24.

**Table 3:** Analysis of immunohistochemical markers (CD68, a-SMA, and fibronectin) of scar tissue in different experimental groups.

**Parameters/Time**		**Experimental Groups**					
		**C**			**C+AT**		***p*-value**
		**Mean**	**SD**		**Mean**	**SD**	
**CD68**							
	7 days	1.71	1.05		1.74	0.89	0.39
	14 days	1.37	1.06		1.50	1.00	0.36
**a-SMA**							
	7 days	1.77	0.59		1.93	0.64	0.18
	14 days	1.95	1.01		1.83	0.85	0.45
**Fibronectin**							
	7 days	1.63	0.70		1.74	0.54	0.47
	14 days	1.99	0.95		2.15	0.78	0.24

C: surgical glue; AT: surgical glue + triamcinolone acetonide; 0: absent; 1: light; 2: moderate; 3: intense;

* *p* < 0.05 when compared to the glue group (contralateral);

SD: standard deviation.

Source: Elaborated by the authors.

The a-SMA is the most common molecular marker to refer to the myofibroblast^25^^-^ 27. However, no significant differences were evidenced between treatments at different times, but it was notable great the presence of myofibroblasts in C+AT (seven days, Table 3), providing an improvement in the healing process in this initial phase of inflammation followed by a decrease, providing an inhibitory effect of the corticosteroid, which corroborates the study by Seibold et al.^17^. During the healing process, myofibroblasts are found throughout the granulation tissue, and their number and distribution over time correlate with the rate of wound contraction^28^^,^ 29, which leads to improved wound healing^30^^,^ 31.

To date, the literature is scarce on *in-vivo* research with the protein fibronectin (glycoprotein produced by fibroblasts, which participates in the extracellular matrix), although several functions are well described and known^32^. In this work, no results were statistically different, but we confirmed a slight increase in this glycoprotein in the C+AT treatment (seven and 14 days, Table 3), and the literature points out its importance in the inflammatory phase, in which fibronectin is capable of opsonizing debris from the extracellular matrix in addition to activating macrophages to that can phagocytose this debris. Fibronectin is an adhesive glycoprotein that plays a crucial role in wound healing, particularly extracellular matrix formation and re-epithelialization^32^. Among the various components of the extracellular matrix, fibronectin is a cell adhesion protein that helps in cell migration and collagen accumulation and, consequently, accelerates wound healing^33^^,^ 34. Wangoo et al.^35^ observed that different concentrations of triamcinolone interfere with the expression of pro collagen and fibronectin, highlighting that lower concentrations of the corticosteroid promote fewer changes in the expression of these proteins, which are also crucial in tissue repair.

## Conclusion

Triamcinolone associated with surgical glue in wound healing confirmed the anti-inflammatory and immunomodulatory effects of the corticosteroid, with less neovascularization and fibrosis, in addition to the remodeling of the extracellular matrix carried out by the balance of myofibroblasts and less dense collagen fibers, reduction of the inflammatory scar phase culminating in tissue regeneration, in addition to possible reduction of side effects of the polymer itself, such as allergic contact dermatitis. The combination of 2-octyl-cyanoacrylate with triamcinolone has promising potential as an adhesive for skin sutures, as there were no changes in the physicochemical characteristics of the compound. Additional future studies will be necessary to substantiate this research, considering the limitations of an experimental animal study, until translational medicine is achieved.

## Data Availability

All data sets were generated or analyzed in the current study.
